# HIV-1 capsid and viral DNA integration

**DOI:** 10.1128/mbio.00212-22

**Published:** 2023-12-12

**Authors:** Richa Dwivedi, Prem Prakash, Bajarang Vasant Kumbhar, Muthukumar Balasubramaniam, Chandravanu Dash

**Affiliations:** 1 The Center for AIDS Health Disparities Research, Meharry Medical College, Nashville, Tennessee, USA; 2 Department of Microbiology, Immunology, and Physiology, Meharry Medical College, Nashville, Tennessee, USA; 3 Department of Biochemistry, Cancer Biology, Neuroscience and Pharmacology, Meharry Medical College, Nashville, Tennessee, USA; 4 Department of Biological Sciences, Sunandan Divatia School of Science, NMIMS (Deemed to be) University, Mumbai, Maharashtra, India; Albert Einstein College of Medicine, Bronx, New York, USA

**Keywords:** HIV, capsid, reverse transcription, nuclear entry, integration

## Abstract

The human immunodeficiency virus type 1 (HIV-1) genome encodes 15 proteins that perform structural, enzymatic, regulatory, and accessory functions. The capsid protein (CA) is the primary structural protein of HIV-1 and plays multiple functions during infection. Early studies predicted that HIV-1 CA mainly protected and delivered the viral genome to the target cell. However, it is now well established that CA plays a critical role after the cellular entry steps of infection. During the early stages, CA promotes reverse transcription of the RNA genome into a DNA copy and the nuclear import/entry step of HIV-1 infection. Emerging evidence also supports the functional role of CA in the post-nuclear entry steps of infection, such as HIV-1 integration. During the late stages of infection, CA coordinates hexameric lattice formation for the assembly of immature virion. CA also regulates theformation of the mature capsid that encases the viral genome and associated factors in the infectious progeny virion. Because of these indispensable roles, HIV-1 CA has emerged as a new and validated target for antiviral drug development. Accordingly, the first CA-targeting drug, lenacapavir (GS-6207), was recently approved to treat certain HIV-1-infected individuals whose viral load cannot be controlled by other antiviral drugs. Still, new and superior CA inhibitors are needed to qualify as part of the front-line antiviral therapy regimens for all infected individuals. The development of such inhibitors requires a clear understanding of CA’s role in HIV-1 infection. In this review, we will describe CA’s role during the early stages of HIV-1 infection, with particular emphasis on post-nuclear entry steps.

**IMPORTANCE:**

HIV-1 capsid protein (CA)—independently or by recruiting host factors—mediates several key steps of virus replication in the cytoplasm and nucleus of the target cell. Research in the recent years have established that CA is multifunctional and genetically fragile of all the HIV-1 proteins. Accordingly, CA has emerged as a validated and high priority therapeutic target, and the first CA-targeting antiviral drug was recently approved for treating multi-drug resistant HIV-1 infection. However, development of next generation CA inhibitors depends on a better understanding of CA’s known roles, as well as probing of CA’s novel roles, in HIV-1 replication. In this timely review, we present an updated overview of the current state of our understanding of CA’s multifunctional role in HIV-1 replication—with a special emphasis on CA’s newfound post-nuclear roles, highlight the pressing knowledge gaps, and discuss directions for future research.

## INTRODUCTION

Human immunodeficiency virus type 1 (HIV-1) is the causative agent of acquired immunodeficiency syndrome (AIDS) (1–3). The HIV/AIDS pandemic has been responsible for ~40 million deaths, with an additional ~39 million people currently infected with the virus (4). HIV-1 infects immune cells, such as T lymphocytes, monocytes, and macrophages, that express the CD4 receptor and CCR5 or CXCR4 chemokine co-receptors (5–11). As a consequence of HIV-1 infection, the number of CD4+ T cells is progressively declining in infected individuals, leading to systemic failure of the immune system, a hallmark of progression to AIDS (12). Fortunately, antiviral therapy (ART) that uses a combination of two to three different drugs has been highly effective in controlling HIV-1 infection and preventing AIDS-related death. Yet, ART is not curative, faces drug resistance, and is often toxic. Thus, a clear understanding of the molecular interactions between HIV-1 and the host will help identify new antiviral drug targets.

HIV-1 infection is broadly categorized into early and late stages. The early stages include the steps of cellular entry, reverse transcription, nuclear entry, and integration. The late stages involve viral RNA transcription, viral protein production, and the assembly, budding, release, and maturation of progeny virions (13–18). HIV-1 infection is initiated when the viral envelope (Env) glycoprotein binds to the CD4 and CCR5/CXCR4 expressed on the plasma membrane of the target immune cells (19, 20). Subsequently, the viral membrane fuses with the plasma membrane of the target cell, resulting in the release of the viral capsid into the cytoplasm (21). The core of the HIV-1 capsid contains two copies of a linear single-stranded (ss) viral RNA genome and a number of other key viral and cellular factors required for the post-entry steps of infection (22–27). The cytoplasmic release of the capsid coincides with the reverse transcription step, where the reverse transcription complex (RTC) containing the viral reverse transcriptase (RT) enzyme starts the conversion of the viral ssRNA into a double-stranded (ds) DNA copy (28). By an unknown mechanism and undefined time, the RTC transitions into a pre-integration complex (PIC). The PIC contains the viral DNA and associated cellular and viral factors, including the viral integrase (IN) enzyme (29). The general and longstanding understanding in the field has been that the PIC containing the reverse-transcribed viral DNA is transported across the nuclear pore complex (NPC) into the nucleus. However, recent findings point to a different mechanism, wherein an intact capsid is competent to enter and the viral RNA undergoes reverse transcription within the nucleus (30, 31). In the nucleus, the PIC-associated IN inserts the viral DNA into hotspots of the host chromosomes (29, 32–34), particularly into transcriptionally active chromatin regions (35). HIV-1 integration establishes a provirus that persists for the lifetime of the host cell and marks the end of the early stages of infection. Notably, the provirus serves as the genetic element for the production of progeny virions during the late stages of infection. Specifically, the provirus is transcribed by the host RNA polymerase II machinery to generate spliced viral mRNAs and non-spliced full-length genomic RNA (36–40). These viral RNAs are exported to the cytoplasm for viral protein production and genome encapsidation (36). Subsequently, the viral proteins and the viral ssRNA genome are trafficked to the plasma membrane of the host cell to assemble the immature virus (13, 41, 42). Finally, through the late steps of budding, release, and maturation, infectious progeny virions are produced to complete the HIV-1 replication cycle (13, 41).

It is well established that both the early and late stages of HIV-1 infection are coordinated by viral proteins. The HIV-1 capsid protein (CA) is the primary structural protein of the virus. The CA was initially thought to be only needed to protect and deliver the viral RNA genome to the target cell. However, it is now recognized that CA plays multiple functions to promote both the early and late stages of infection. In this review, we will focus on the role of HIV-1 CA during the early stages of infection. Particularly, we will describe CA’s role in post-nuclear entry steps of infection and highlight the current knowledge gaps in CA’s intranuclear role. For clarity, we will use the terms “HIV-1 replication complex” or “viral replication complex” for RTC, PIC, and other complexes formed during the course of infection. It should also be noted that the structural and compositional details of functional PICs and RTCs, either in an infected cell or as extracted biochemical complexes, are poorly understood. Although both complexes share a number of common factors, the spatiotemporal staging of the RTC and the PIC in an infected cell relative to viral entry also remains unsettled. Yet, RTCs and PICs are functionally distinct viral complexes as they perform the unique activities of reverse transcription and viral DNA integration, respectively, both *in vitro* and in infected cells. Therefore, we will refer to RTCs and PICs as functionally distinct HIV-1 replication complexes.

### HIV-1 CA organizes into a unique capsid geometry

**FIG 1:**
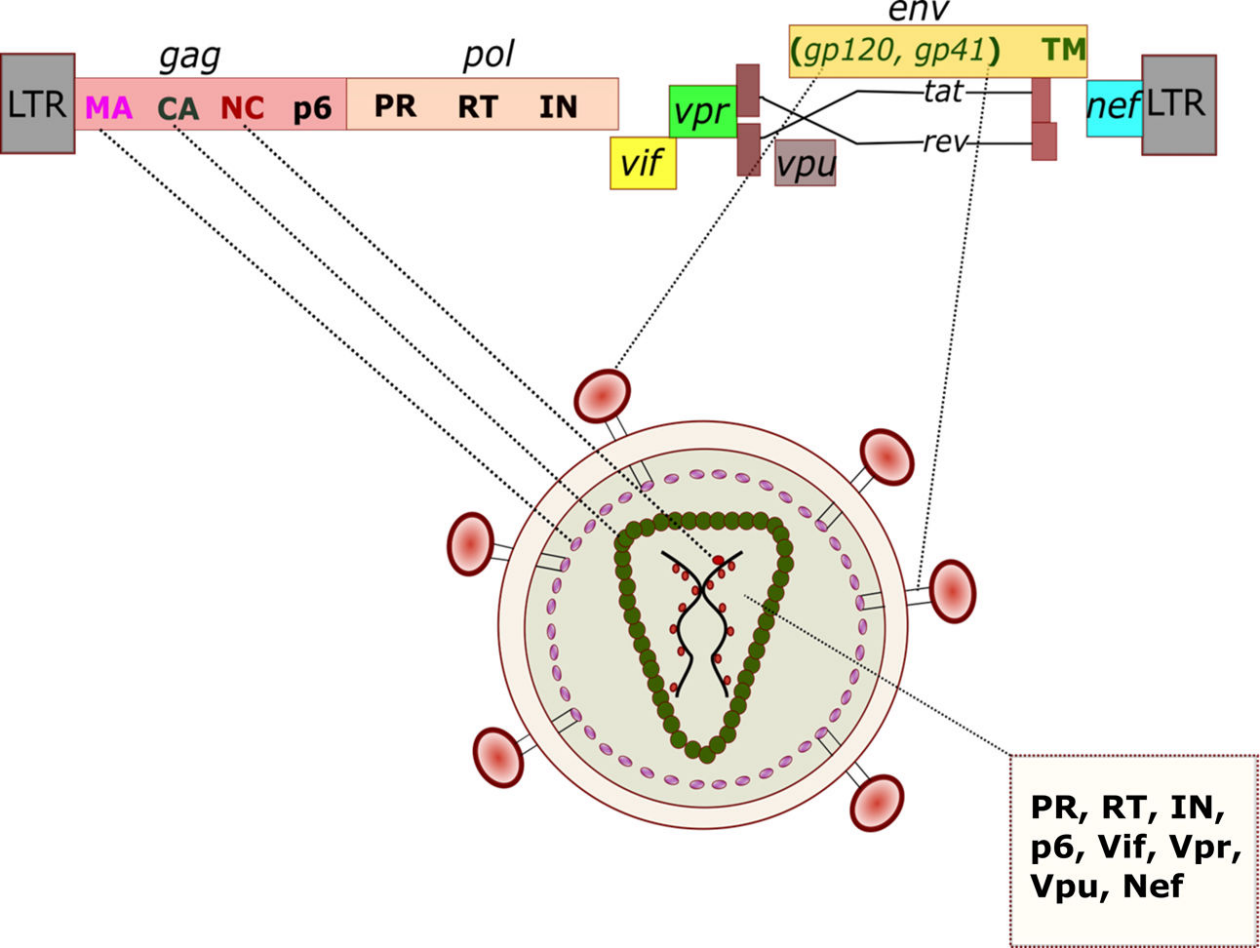
A cartoon depiction of the HIV-1 particle and a schematic representation of the viral genomic map. The mature particle contains two genomic RNA molecules (shown here as black curved lines inside the green-colored capsid) that encode nine open reading frames and contain long terminal repeats (LTRs) at both the 5′ and 3′ ends. The RNA genome encodes viral polyprotein precursors (Gag, Gag-Pol) or, after splicing, viral envelope (gp160), regulatory (Tat, Rev), and accessory proteins (Nef, Vpr, Vpu, Vif). The HIV-1 proteins are generated after the integration of the reverse-transcribed viral DNA, followed by transcription into viral mRNAs. The HIV-1 PR-mediated processing of the Gag into MA, CA, NC, and p6, and the processing of Gag-Pol into RT and IN proteins within the released immature virus particle trigger the assembly of the fullerene cone-shaped capsid and the generation of the mature infectious HIV-1 particle (virion). The capsid contains RT, IN, PR, Vif, Vpr, Vpu, Nef, and HIV-1 genomic RNA dimers coated with NC. The host cell protease-mediated processing of the viral envelope polyprotein gp160 in the producer cell yields trimeric complexes of heterodimers of gp120 and gp41 that are embedded in the host cell membrane enclosing the virus.

**FIG 2:**
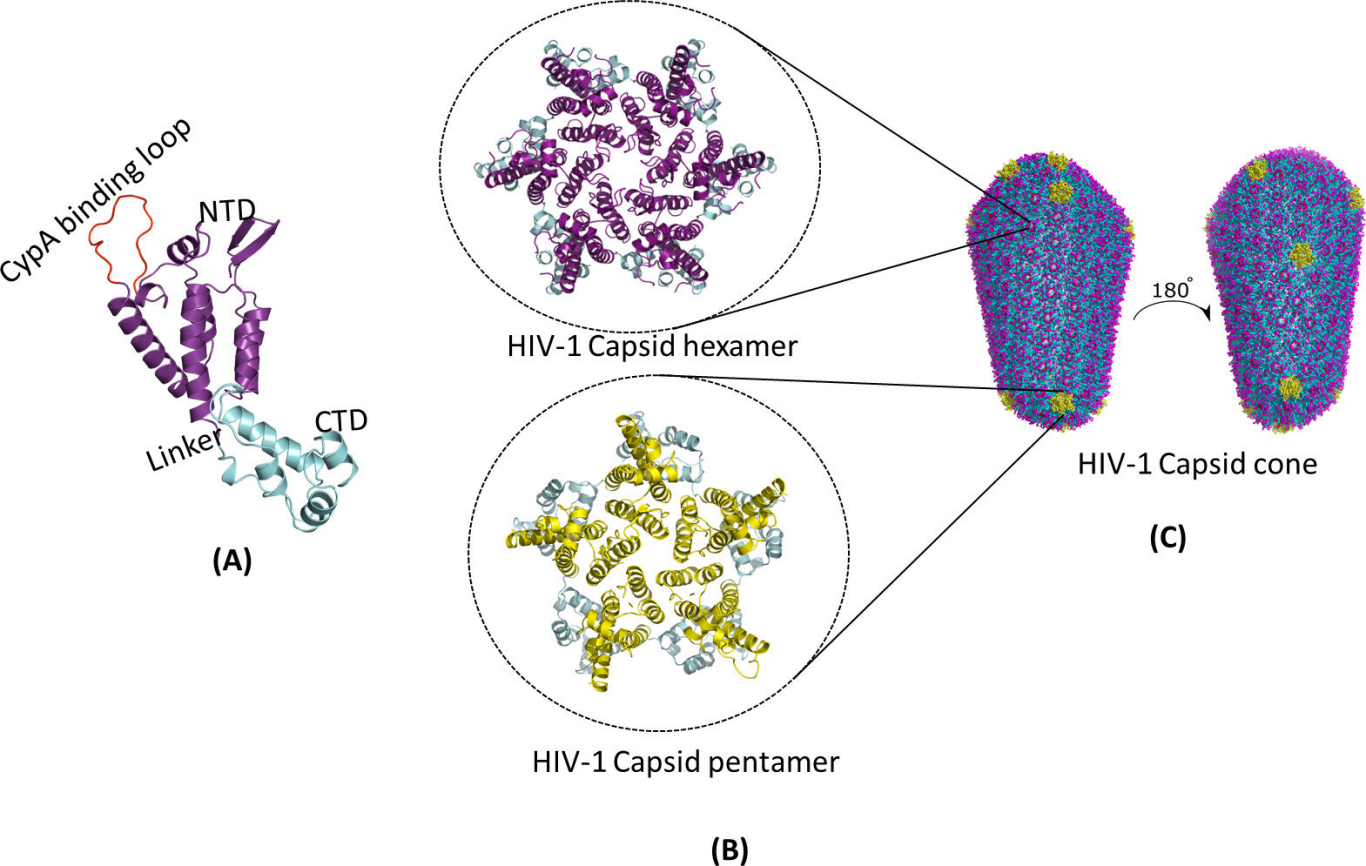
HIV-1 CA oligomerization into a conical lattice of hexamers and pentamers. (A) Ribbon diagram of the CA monomer (PDB ID: 4XFY) with the N-terminal domain (NTD, purple), a linker region, the C-terminal domain (CTD, cyan), and the unstructured cyclophilin A (CypA)-binding loop (red). (B) A zoomed-in view of the CA hexamers and pentamers that constitute the HIV-1 capsid fullerene cone. In the upper inset, the top view of the CA hexamer (PDB ID: 4XFY) is depicted with the NTD (purple) oriented toward the anterior and the CTD (cyan) positioned toward the posterior. In the lower inset, the top view of the CA pentamer (PDB ID: 3P05) is shown with the NTD (yellow) oriented toward the anterior and the CTD (cyan) oriented toward the posterior. (C) The entire HIV-1 capsid cone (PDB ID: 3J3Y) is composed of CA hexamers (purple) and CA pentamers (yellow). A 180° rotation view depicts the positioning of the pentamers on the capsid surface.

HIV-1 replication is dependent on a small number of viral proteins encoded within an ~9.7 kb positive-sense ssRNA genome (43). The HIV-1 genome contains several cis-acting structural elements and 9 open-reading frames (ORFs) to encode 15 viral proteins (Fig. 1) (44). The *gag* ORF encodes the Gag polyprotein precursor for four structural proteins: matrix (MA), CA, nucleocapsid (NC), and p6. The *pol* ORF encodes the polyprotein for three viral enzymes: protease (PR), RT, and IN. Similarly, the *env* ORF codes for an Env polyprotein containing glycoprotein 120 (gp120) and glycoprotein 41 (gp41). The remaining six ORFs encode the following: (i) the accessory proteins such as virion infectivity factor (Vif), viral protein R (Vpr), viral protein U (Vpu), and negative factor (Nef), and (ii) the regulatory proteins such as trans-activator of transcription (Tat) and regulator of expression of virion (Rev). The viral Gag and GagPol polyprotein precursors are cleaved by the viral PR enzyme, whereas the Env polyprotein precursor is processed by the host cell protease furin to release the respective mature functional HIV-1 proteins. Together, these 15 viral proteins perform all the necessary structural, enzymatic, regulatory, and accessory functions to coordinate and promote both the early and late stages of HIV-1 infection. Particularly, the HIV-1 CA monomer, the protein of interest in this review, released from the Gag precursor by PR-mediated cleavage, serves as the building block of the conical viral capsid (Fig. 1 and 2).

HIV-1 is a spherical enveloped particle with reported diameters ranging from ~120 to 200 nm (Fig. 1) (45). The virion has an outer lipid bilayer membrane that surrounds a cone shaped capsid (22, 46). The capsid is composed of ~1,200–1,500 copies of CA monomers arranged into ~200–250 hexamers and 12 pentamers (Fig. 2). The distinctive fullerene curvature of the HIV-1 capsid is formed by the precise distribution of seven CA pentamers at the broad end (~60 nm in diameter) and five CA pentamers at the narrow end (~20–30 nm in diameter) of the cone. Structurally, the HIV-1 CA monomer is an α-helical protein and has two distinct domains, an N-terminal domain (CA-NTD) and a C-terminal domain (CA-CTD), connected by a flexible linker (Fig. 2). The CA-NTD contains seven α-helices (α1–α7), an extended cyclophilin A (CypA)-binding loop, and a β-hairpin. The CA-CTD consists of four α-helices (α8–α11), a short 3_10_-helix, and the major homology region. Notably, a number of specific CA-CA molecular interactions determine the assembly and stability of the capsid. The NTD-NTD contacts between CA monomers stabilize the hexamers and pentamers and form a central pore gated by the β-hairpin. The NTD-CTD contacts between adjacent CA monomers further stabilize the hexamers and pentamers. The CTD-CTD contacts participate in dimeric and trimeric interactions between individual hexamers and pentamers. These molecular CA interactions create at least three known interfaces for host factor binding (47–60). These include a hydrophobic binding cleft created within a hexamer, the R18 pore at the center of the hexamer, and a CypA-binding loop on each CA subunit. Additionally, the restriction factor tripartite motif 5α (TRIM5α) forms a large hexagonal lattice on the capsid shell spanning six CA hexamers (56, 57, 61). Importantly, the highly ordered structure and integrity of the HIV-1 capsid are essential for viral infectivity.

### CA regulates both the early and late stages of HIV-1 infection

**FIG 3:**
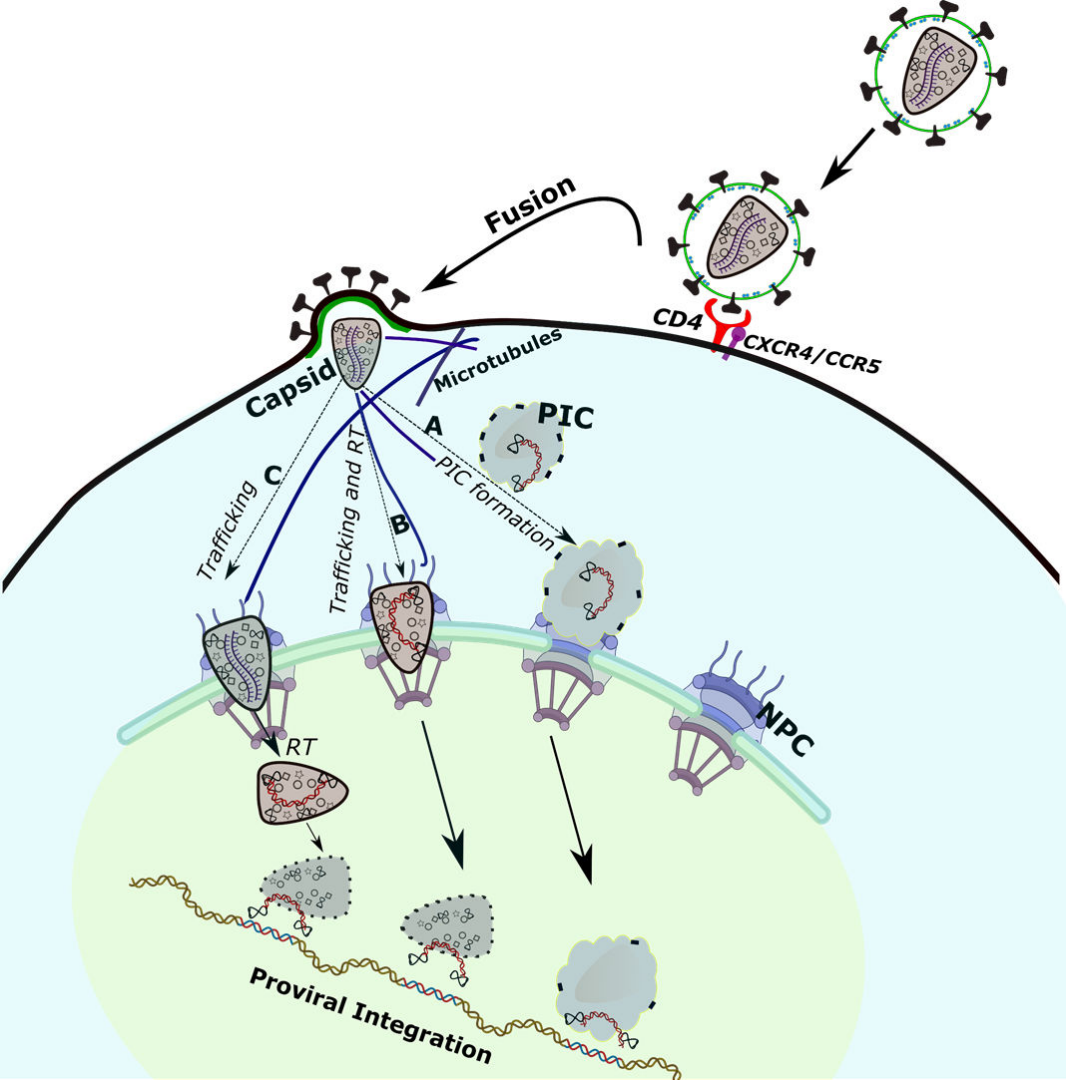
Illustration of the canonical and emerging models of the early events of HIV-1 replication culminating in viral integration. Sequential binding of the HIV-1 envelope glycoprotein gp120 to the CD4+ receptor and to one of the co-receptors (CCR5 or CXCR4) leads to the fusion of the viral and cellular membranes and the release of the capsid into the cytoplasm. The capsid contains two copies of the linear ssRNA viral genome as well as certain viral and cellular factors, and it is trafficked by the host microtubule machinery through the cytoplasm toward the nucleus. While the canonical and emerging models of the early events of HIV-1 replication concur that the reverse transcription begins within the confines of the capsid, the models differ on the spatiotemporal staging of the reverse transcription and the subsequent steps. In the canonical sequence of events (A), reverse transcription occurring within the capsid in the cytoplasm leads to two concurrent outcomes: the generation of the PIC and the uncoating of the capsid. The PIC contains the viral dsDNA complexed with multimers of the viral IN, as well as other viral and cellular factors. The PIC is then imported via the NPC into the nucleus, wherein the IN inserts the viral DNA into the chromosomal DNA (integration). An alternate version of early events proposes that (B) reverse transcription is ongoing or completed within the capsid during its cytoplasmic trafficking to the NPC, and the intact or partially intact capsid is imported into the nucleus, where further loss of the capsid’s structural integrity during its intranuclear trafficking enables viral DNA integration into the chromosomal DNA. In another emerging version of early events (C), reverse transcription is initiated only upon nuclear import of the intact capsid and is completed during intranuclear trafficking, which coincides with the loss of the capsid’s structural integrity that enables viral DNA integration.

Initially, it was predicted that HIV-1 CA protected and delivered the viral genome into the cytoplasm of the target cell (Fig. 3). However, it is now established that CA is a multifunctional protein and regulates the steps of reverse transcription, nuclear entry, integration, viral assembly, and maturation (62–67). For instance, HIV-1 CA is functionally linked to the reverse transcription process, where the viral ssRNA genome is converted into a dsDNA copy by the RTC in a step-wise manner. Although the exact mechanism by which CA promotes HIV-1 reverse transcription is not fully understood, it is predicted that the capsid provides a suitable and efficient microenvironment for the RTC to complete viral DNA synthesis. CA also directly regulates the nuclear entry of the PIC, which traffics the reverse-transcribed viral DNA into the nucleus of the target cell. Interestingly, in early studies, a number of viral proteins, including IN, MA, and Vpr, were reported to possess a nuclear localization signal and were implicated in HIV-1 nuclear import (68–71). However, CA has emerged as the only viral protein responsible for the cytoplasmic trafficking and the nuclear import of HIV-1 replication complexes (64, 65). For example, the microtubule network proteins dynein and kinesin of the host cell facilitate HIV-1 trafficking toward the NPCs (72, 73). Several dynein subunit proteins were subsequently reported to be involved in HIV-1 trafficking (74–76). Particularly, the dynein adapter protein bicaudal D2 (BICD2) was shown to bind HIV-1 capsid and regulate its trafficking (77, 78). Interestingly, disruption of this trafficking mechanism leads to the binding of the capsid by Kinesin-1 via the adapter protein FEZ1, which initiates anterograde movement of the capsid along the same microtubule (79–81). Structural and biochemical data also highlight that CA provides binding interfaces for a number of other host factors to facilitate the nuclear entry of HIV-1. For example, the host factor CypA binds to CA to promote both reverse transcription and nuclear import of the virus (82). Host proteins such as Nucleoporin 358 (Nup358), Nucleoporin 153 (Nup153), and cleavage and polyadenylation specificity factor 6 (CPSF6) also bind to CA to promote HIV-1 nuclear entry (52, 82–85). Emerging evidence also suggests a functional role of CA-host factor interaction in post-nuclear entry steps, including HIV-1 integration. For instance, CA-CPSF6 interaction regulates HIV-1 integration targeting specific regions of the host genome (86, 87). Finally, CA also coordinates key steps during the late stages of HIV-1 infection (13, 41). For example, during viral assembly, CA is involved in hexameric lattice formation in the immature virion (88–94) and also regulates the formation of the cone-shaped mature capsid in the progeny virion during the maturation step (25, 26, 95–100).

Because CA plays essential roles at multiple steps of HIV-1 infection, it has emerged as a validated and highly attractive target for antiviral drug development. Recently, the first CA-targeting drug, lenacapavir (GS-6207), was approved by the United States Food and Drug Administration for the treatment of certain HIV-1-infected individuals whose viral load cannot be controlled with other antiviral treatment options (101–104). Although narrow in clinical application, approval of GS-6207 marks a major milestone for CA-based antiviral therapy. However, new and superior CA inhibitors are urgently needed to become part of the front-line treatment for all HIV-1-infected individuals. The development of such inhibitors is dependent on a clear understanding of the mechanisms by which CA controls both the early and late stages of HIV-1 infection. In this review, we will focus on CA’s role in post-nuclear entry steps of HIV-1 infection, since several excellent reviews of CA’s role in reverse transcription and nuclear entry (72, 105–113) as well as virus assembly, budding, and release (14, 18, 25, 107, 114) have been published in recent years.

### CA’s role in post-nuclear entry steps of HIV-1 infection

After the nuclear entry step, HIV-1 DNA is integrated into transcriptionally active regions of the host chromosomes (29, 32–34). HIV-1 IN is the viral protein primarily responsible for carrying out HIV-1 DNA integration. First, the PIC-associated IN carries out the 3′-end processing step, where the 3′-GT dinucleotides are cleaved from both the viral DNA ends to generate recessed 3′-OH-containing termini (29, 34, 115, 116). Subsequently, the 3′-processed viral DNA is inserted into the host DNA by a strand transfer step, where the 3′-OH of the viral DNA carries out a nucleophilic attack on the phosphate backbone of the host DNA (29, 34, 115, 116). Finally, the two unpaired nucleotides of the 5′-end of the viral DNA are removed, and the five nucleotide gaps at the junction of the inserted viral DNA ends are filled by the cellular repair enzymes (29, 34, 115, 116). The resulting provirus becomes a stable genetic element of the host cell, and its expression leads to the production of progeny virions during the late stages of infection.

A growing body of literature suggests that CA affects post-nuclear entry steps of HIV-1 infection, including viral DNA integration. For instance, CA is associated with nuclear HIV-1 replication complexes. Specific mutations in CA also affect HIV-1 integration patterns in the host genome. Similarly, small-molecule inhibitors that specifically target CA inhibit HIV-1 integration and alter integration targeting. Notably, biochemical evidence demonstrates that CA regulates the DNA integration activity of HIV-1 PICs *in vitro*. Finally, the recent reports showing that the entire HIV-1 capsid enters the nucleus of an infected cell strongly suggest a direct role of CA in the post-nuclear entry steps of infection. We will discuss these lines of evidence to pinpoint a functional and/or direct role of HIV-1 CA in the nucleus of an infected cell.

#### Studies suggest a lack of CA in functional HIV-1 PICs

HIV-1 replication complexes, such as RTC and PICs containing reverse transcription activity and DNA integration activity, respectively, can be extracted from infected cells. For instance, PICs have been extracted from both the cytoplasm and nucleus of acutely infected cells (117, 118). These PICs retain the activity to insert the viral DNA into a range of exogenous DNA substrates (e.g., linear DNA, plasmid DNA, nucleosome-bound DNA, and isolated chromatin) *in vitro* (117, 118). Therefore, PIC studies have been instrumental in understanding the mechanisms of HIV-1 integration, identifying PIC-associated viral and cellular factors, and developing IN inhibitors. Accordingly, PIC studies have also been instrumental in identifying a functional and direct role for CA in HIV-1 DNA integration (117, 119–137).

Early biochemical studies suggested that CA is completely shed from HIV-1 PICs and may not be directly involved in the post-nuclear entry steps of infection. Farnet and Haseltine were the first to extract HIV-1 PICs from the cytoplasm of acutely infected cells (133). They demonstrated that the PIC can insert the viral DNA into dsDNA substrates but not into ssDNAs. Fractionation studies of the extracted PICs showed that the peak levels of viral DNA contained less than 1% of the total eluted protein. However, fractions with peak levels of viral DNA had no detectable amount of CA. Since the viral DNA molecules present in the peak fractions were capable of integrating into target DNA *in vitro*, it was concluded that most of the CA is not complexed or dissociated from the PIC without a loss of integration activity. In a follow-up study, Farnet and Haseltine also compared cytoplasmic PICs from cells infected with HIV-1 or murine leukemia virus (MLV) (134). Similar to their previous study, they failed to detect HIV-1 CA in the HIV-1 PIC extracts. Interestingly, MLV CA was found to be associated with the MLV PIC preparations. Thereafter, Bukrinsky et al. extracted functional HIV-1 PICs from both the cytoplasm and nucleus of acutely infected cells (137, 138). These authors also reported that CA is not associated with the PICs extracted from either the cytoplasmic or nuclear compartments. The lack of CA in the PICs was also reported by Karageorgos et al. (139). In contrast to the previous studies that used cell-free virions for infection, this study analyzed PICs generated by cell-to-cell transmission. Following cytoplasmic and nuclear PIC extraction, sucrose gradient fractions containing the peak of viral DNA were subjected to immunoprecipitation to detect viral proteins. Results from these studies showed a lack of CA in the PICs of the cytoplasmic or nuclear fractions. Subsequently, Miller et al. also analyzed the composition of cytoplasmic HIV-1 PICs by gel filtration chromatography (136). Interestingly, PICs fractionated in low-salt conditions (150 mM KCl) retained full integration activity, whereas the activity was lost in higher-salt conditions (600 mM KCl). Interestingly, a small amount of CA was detected in PICs fractionated in 150 mM KCl but not in 600 mM KCl. Thus, it was concluded that CA is loosely associated with the PIC and may not play an important role in viral DNA integration. Finally, Fassati and Goff extracted HIV-1 replication complexes from HeLa cells as a function of time, starting from viral entry to the completion of reverse transcription (140). Analyses of the cytoplasmic complexes indicated that the newly synthesized viral DNA sedimented at a higher density without any CA. Interestingly, CA was detected in fractions having a buoyant density, suggesting that most CA was dissociated from HIV-1 replication complexes early after viral entry.

Collectively, these studies suggested that CA is largely dissociated from HIV-1 PICs, either in the cytoplasm or at the nuclear pore. Thus, it was predicted that CA may not be directly and/or functionally involved in the post-nuclear entry steps of HIV-1 infection. It should be noted that the approaches used in these early studies lacked the sensitivity to detect a very small amount of CA in the viral replication complexes. Therefore, the possibility that a small amount of CA could be associated with the functional PIC could not be ruled out.

#### Detection of CA with HIV-1 PICs in the cytoplasm

Although biochemical studies could not pinpoint a direct role of CA in PIC function, imaging and genetic studies detected CA associated with HIV-1 replication complexes at or near the nuclear pores (73, 141). These studies also reported the presence of CA in viral replication complexes in the nucleus of infected cells (131), suggesting that CA is potentially involved in the post-nuclear entry steps of HIV-1 infection. First, McDonald et al. studied the replication complexes of GFP-labeled HIV-1 particles in adherent cells (73). They detected two distinct viral complexes after cellular entry, one lacking CA and the other containing significant amounts of CA. They concluded that the CA remains associated with the replication complex from the initiation of reverse transcription to the maturation of the PIC. These observations were consistent with a seminal study by Forshey et al. demonstrating that the HIV-1 core remained intact for some period to promote reverse transcription (66). For instance, CA mutants with altered core stability (either hyper-stable or unstable) were impaired in infectivity. Most CA mutants were competent for cellular entry but were blocked for reverse transcription. Interestingly, two CA mutants, P38A and Q63A/Q67A, were capable of efficient reverse transcription even though the particles were much less infectious. In a follow-up study, Dismuke and Aiken demonstrated that the Q63/67A mutant virus is impaired for nuclear entry (67). Cytoplasmic PICs extracted from cells infected with the Q63/67A mutant retained lower integration activity and higher levels of CA than the wild-type PICs. Thus, it was suggested that an elevated level of PIC-associated CA inhibits nuclear entry of the mutant PIC and/or integration. To the best of our knowledge, this is the first report suggesting a direct role of CA in PIC function, although *in vitro*. Thereafter, Arhel et al. examined the fate of HIV-1 CA in an infected cell by immunofluorescence confocal microscopy (131). They detected CA signals immediately after viral entry that shifted from the plasma membrane to the nuclear membrane. Notably, a perinuclear ring of CA was detected as early as 2 hpi and up to 24 hpi, which was progressively lost between 24 and 48 hpi. Since the perinuclear CA colocalized with the viral DNA, it was hypothesized that HIV-1 replication complexes at the cytoplasmic face of the nuclear membrane contained CA. Interestingly, scanning electron microscopy of the nuclear surface of infected cells also showed conical capsid-like structures near nuclear pores. Thus, it was predicted that CA uncoating occurs at the nuclear pore upon completion of viral DNA synthesis. However, the fate of the capsids during and/or after nuclear entry could not be tracked using scanning electron microscopy. The presence of CA in viral replication complexes was also reported by Lelek et al. using super-resolution microscopy (141). Cytoplasmic clusters of CA were detected with a size comparable to the intact capsid, suggesting that cytoplasmic viral complexes contain some form of intact capsid. Collectively, these studies provided evidence that cytoplasmic replication complexes (RTC and/or PICs) contain detectable levels of CA. These observations also supported the model that CA is the key determinant of HIV-1 nuclear entry (63, 65, 142–148). However, the lack of CA in the nucleus implied that HIV-1 replication complexes undergo complete CA uncoating prior to or during the nuclear entry step of infection (Fig. 3).

Recently, Christensen et al. reported a cell-free system that recapitulated the sequential processes of reverse transcription and concerted integration by viral cores (149). Endogenous reverse transcription within viral cores, released by gentle lysis of purified virions, was initiated by the addition of deoxynucleotide triphosphates, ribonucleotide triphosphates, optimized buffer, inositol hexakisphosphate, and (optional) cell extract. Notably, these reactions generated canonical reverse transcription products in the predicted temporal order, and the synthesis of viral dsDNA triggered localized capsid disassembly. Furthermore, the addition of cell extracts led to the concerted integration of the reverse-transcribed viral DNA into an exogenous target DNA. Importantly, inhibition of reverse transcription blocked viral core disassembly, and hyper-stabilization of viral cores impaired viral integration. These studies indicated that reverse transcription is critical for viral core disassembly and that partial or complete disassembly of the viral core is essential for integration to proceed.

Finally, we have reported the functional association of CA in cytoplasmic PICs using two independent approaches (123). We carried out partial purification of cytoplasmic PICs through sucrose gradient fractionation. Integration activity measurements *in vitro* concurrent with the presence of viral DNA enabled us to identify functional PICs. Furthermore, detection of CA in the same fractions containing integration activity and viral DNA demonstrated that CA is associated with the functional PICs. An immunoprecipitation assay with a monoclonal CA-specific antibody also confirmed the presence of CA in these cytoplasmic PICs. Interestingly, we also observed that the integration activity of these PICs is reduced in the presence of a CA-specific monoclonal antibody. Collectively, these observations strongly support a direct link between CA and PIC-associated integration activity.

#### Association of CA with HIV-1 PICs in the nucleus

The presence of HIV-1 CA in the nucleus of an infected cell was first reported by Zhou et al. (150). In this study, a time-course of CA accumulation was probed in TNPO3-depleted cells and was compared with TNPO3-expressing wild-type cells after inoculation with HIV-1 GFP particles. In TNPO3-depleted cells, trace amounts of nuclear CA were detected at 6 hpi, which progressively increased up to 24 hpi. Interestingly, the timing of CA nuclear accumulation mirrored the kinetics of viral integration. Therefore, it was proposed that after nuclear entry, TNPO3 promotes PIC maturation, where CA associated with nuclear PIC was removed to facilitate HIV-1 DNA integration. The presence of nuclear HIV-1 CA in an infected cell was also reported by Peng et al. (142). By click-labeling the nascent viral DNA synthesized during reverse transcription, this study detected CA in cytoplasmic replication complexes but not in the nuclear PICs in a HeLa-derived reporter cell line. In contrast, in infected primary human monocyte-derived macrophages (MDMs), almost all nuclear PICs contained CA. A number of subsequent studies also demonstrated the presence of CA in the nuclear replication complexes of HIV-1 using a variety of different approaches (63, 143, 145, 146, 148).

Chin et al. used a branch-chain DNA variant of fluorescence *in situ* hybridization in combination with immunolabeling of CA to visualize HIV-1 replication complexes (143). HIV-1 CA was detected in the nuclear replication complexes of HeLa cells, in contrast to the studies of Peng et al. (142). Nuclear CA was also detected in infected U20S cells and MDMs and was associated with the viral DNA, suggesting a functional role of CA in the nucleus of infected cells. Hulme et al. combined a capsid integrity assay with CA inhibitors and high-resolution structured illumination microscopy to monitor the fate of CA in the cytoplasm and nucleus of HIV-1-infected cells (63). They observed that the viral complexes in the cytoplasm, nuclear membrane, and nucleus contained a range of CA levels. By systematically analyzing the distribution of CA levels in these complexes, they predicted that the majority of CA uncoats in the cytoplasm, and a subset of CA remains associated with the viral complex after uncoating. They further suggested that the uncoating of the residual CA from the nuclear PIC facilitates post-nuclear entry steps of infection, in accordance with the studies of Zhou et al. (150). Accordingly, Burdick et al. reported the presence of lower amounts of CA with viral complexes in the nucleus (146). They suggested that the viral cores undergo extensive CA dissociation and/or conformational rearrangements as a prerequisite for nuclear import or entry. Stultz et al. also demonstrated the presence of CA in viral DNA-containing viral complexes in both the cytoplasm and nucleus of infected MDMs (145). Interestingly, this study showed that faster reverse transcription in MDMs, induced by Vpx-mediated SAMHD1 degradation, accelerated core uncoating concurrent with decreased colocalization of CA with the nuclear PICs. Bejarano et al. also used the Vpx-mediated SAMHD1 degradation approach to study the effects of reverse transcription kinetics on core uncoating, nuclear entry, and intra-nuclear steps (151). Similar to previous reports, CA was detected in the PIC at all subcellular localizations, including in the nucleus of the MDMs. Notably, there was no apparent lag phase between the completion of reverse transcription and integration in MDMs, alluding to the model that reverse transcription could be completed in the nucleus of MDMs just before viral DNA integration. Importantly, recent studies suggest that in macrophages, the incoming viral RNA genomes can enter and be reverse transcribed within the nucleus (30, 31). Francis and Melikyan also visualized early steps of HIV-1 infection using virus particles labeled with CypA-DsRed as a decoy for detecting CA in replication complexes (148). In this study, virtually all replication complexes that docked at the NPC contained CypA-DsRed, implying the presence of CA. Notably, loss of CypA-DsRed (and, by extension, removal of CA) correlated with nuclear entry of the PIC. Interestingly, induction of early uncoating in the cytoplasm led to proteasomal degradation of the viral replication complex. Thus, it was suggested that cores must retain a significant amount of CA to be protected in the cytoplasm and dock at the nuclear pore. Furthermore, accelerated loss of the majority of CA at the nuclear pore was a prerequisite for the nuclear import of HIV. Nevertheless, a small amount of CA was detected in nuclear viral replication complexes. It was demonstrated that the interaction of host factors with the nuclear CA was required to facilitate nuclear penetration and integration site preference. Bonisch et al. also found CA in the nucleus of infected HeLa-derived adherent cells (152). However, the function of the nuclear CA in this study was unclear since it was not part of integration-competent complexes. Finally, Selyutina and Diaz-Griffero developed a protocol wherein they fractionated lysates from HIV-1-infected cells into cytosolic and nuclear fractions and used western blotting to detect CA in both fractions (153). Although the method employed an additional western blotting step to verify the purity of the fractions, the presence of the nuclear envelope in the nuclear fraction could carry over any CA docked at the cytoplasmic side of the NPC. Nevertheless, these expansive studies indicated that CA, although at a lower level, is associated with the HIV-1 replication complexes in the nucleus of infected cells, particularly in transformed adherent cells and primary MDMs.

The development of cutting-edge imaging and microscopy approaches allowed the detection of nuclear CA in mostly adherent cells. However, the presence of nuclear CA in HIV-1-infected T cells remained unclear due to the inherent challenges of imaging suspension cells. To address this technical challenge, Zila et al. used fluorescence and super-resolution microscopy to probe CA levels in the cytoplasmic and nuclear complexes in T cell lines (154). They reported the loss of the majority of CA from HIV-1 replication complexes immediately after cellular entry into these cells. However, the cytoplasmic complexes retained a residual amount of CA that remained unchanged until the complexes reached the nuclear pore. Interestingly, the nuclear PICs were largely devoid of detectable levels of CA in these T cell lines. In contrast, Blanco-Rodriguez et al. showed that viral CA is associated with the nuclear PICs of HIV-1-infected T cells (126). Using an approach to directly label HIV-1 DNA combined with correlative light and electron microscopy, the organization of CA in replication complexes was probed before, during, and after nuclear translocation in both HeLa cells and primary T lymphocytes. They proposed that the CA-associated HIV-1 replication complexes undergo substantial remodeling at the NPC since intact capsids were not detected in the nucleus. Interestingly, CA complexes organized in a pearl necklace-like shape were observed on the nuclear side of the NPC. In another report, Dharan et al. reported that CA uncoating occurs in the nucleus of HIV-1-infected T cell lines (155). Using a clever approach to the inducible NPC blockade assay, it was demonstrated that nuclear import precedes the completion of reverse transcription and uncoating. Thus, it was hypothesized that the completion of CA uncoating occurs after the nuclear entry step, implying the presence of nuclear CA in infected T cell lines. Subsequently, using 3D correlative light and electron microscopy, Müller et al. showed that HIV-1 complexes are associated with CA in the nucleus of infected cells, including primary CD4+ T cells (156). They also reported that CA dissociated from the nuclear complexes, providing further support for a nuclear CA uncoating event. The completion of CA uncoating in the nucleus of T cell lines as well as HeLa and THP-1 monocytic cell lines was also reported by Burdick et al. (157). Using GFP-CA-labeled HIV-1 particles combined with live-cell microscopy, viral cores were tracked in infected cells until integration and proviral DNA transcription. The results demonstrated that viral cores in the nucleus are nearly intact and reverse transcription is completed in the nucleus before uncoating, in accordance with the findings of Dharan et al. (155). In a follow-up study, Li et al. described further that intact HIV-1 capsid docks at the NPC, enters the nucleus, and quickly transports to the site of integration (158). Surprisingly, the intact capsid could be detected in the nucleus for several hours, followed by a rapid loss of CA (akin to uncoating) just minutes before viral DNA integration (158). Collectively, these studies provided strong evidence that CA is associated with the nuclear complexes of HIV-1 in infected T cells and cell lines. Most importantly, these studies also supported a new model that (i) intact or nearly intact HIV-1 capsid enters the nucleus of an infected cell and (ii) capsid uncoating as well as completion of reverse transcription are intranuclear events. Finally, Zila et al. suggested that the narrow end of the HIV-1 capsid docks onto nuclear pores, and intact capsids penetrate the central channel of the NPC (159). This study also indicated that the central channel of the NPC is physically compatible with translocating the intact HIV-1 capsid. Interestingly, upon departure from the NPC central channel, disrupted HIV-1 capsids were detected in the nucleus of the infected cells. Therefore, it was predicted that HIV-1 capsids may not disassemble into individual subunits during uncoating but are morphologically altered during nuclear entry to release the PIC in the nucleus of the infected cell. This capsid disruption model of uncoating is supported by a recent study by Christensen et al., demonstrating that HIV-1 capsids are partially broken upon completion of viral DNA synthesis in purified cores *in vitro* (149). Collectively, these studies provide strong evidence that CA is associated with the nuclear PICs and also suggest that the removal of CA from these PICs is a requirement for HIV-1 integration.

### Potential role of nuclear HIV-1 CA

Despite the growing evidence that CA is present in the nucleus, associated with the nuclear PIC, and removed prior to integration, a functional and/or direct role of CA in the nuclear HIV-1 replication complex remains speculative. Additionally, there are no published reports describing the physical interaction between CA and PIC-associated viral factors such as IN and viral DNA that are required for HIV-1 integration. Nevertheless, it can be envisioned that the nuclear HIV-1 replication complex-associated CA is most likely required for host factor binding, protection of the PIC-associated viral DNA from cellular sensing, nuclear completion of reverse transcription, and viral DNA integration.

#### Host factor binding and integration targeting

The strongest evidence for an intra-nuclear role of HIV-1 CA is provided by studies of host factors that promote early steps of infection. A number of host factors specifically bind to CA associated with the HIV-1 replication complexes in the cytoplasm and nucleus of an infected cell. Interestingly, disruption of CA and host-factor binding alters HIV-1 DNA integration into the host chromosomes.

HIV-1 DNA is preferentially integrated into gene-dense regions and transcriptionally active genes (160). Furthermore, active chromatin with histone modifications such as H4K16ac, H3K36me3, and H3K4me1 is positively associated with HIV-1 integration targeting (161, 162). In contrast, HIV-1 integration is disfavorable in heterochromatin regions with H3K9me3, H3K27me3, and lamina-associated domains (161, 162). HIV-1 integration is also highly favored in speckle-associated domains (35) and genic regions containing recurrent integration genes (86, 161). Although alterations in HIV-1 integration targeting were first reported for the IN-targeting host factor LEDGF/p75 (163), CA-binding host factors such as CypA, Nup358, Nup153, and CPSF6 have also been reported to influence integration targeting (85, 164–168). For instance, a decrease in HIV-1 integration into gene-dense regions was observed in both Nup358- and Nup153-depleted cells (169–171). There is also evidence that Nup98 binds to HIV-1 CA, and depletion of this Nup protein alters integration targeting (171). However, the leading CA-binding host factor implicated in HIV-1 integration targeting is CPSF6. For instance, CPSF6 knockdown dramatically reduced HIV-1 integration targeting into gene-dense regions (86, 87). Notably, tandem knockout of CPSF6 and LEDGF demonstrated that CPSF6 plays a more dominant role than LEDGF in directing HIV-1 integration into gene-dense regions (87). Thus, it has been suggested that CA-CPSF6 binding directs HIV-1 DNA to actively transcribed chromatin and IN-LEDGF binding directs integration into gene bodies. Accordingly, disrupting CA-CPSF6 interaction through CA mutations resulted in the loss of integration targeting into gene-dense regions (169, 170). Interestingly, disrupting CA-CypA interaction through specific CA mutations increased targeting of HIV-1 integration into gene-dense regions (35, 85, 172), in contrast to results obtained with other CA-binding host factors. It should be noted that among these CA-binding host factors, CPSF6 is the only protein known to engage with the nuclear HIV-1 replication complex. Thus, the mechanism by which host factors that engage with the cytoplasmic replication complex influence HIV-1 integration targeting is not fully understood. Nevertheless, we have recently reported a functional role of CypA in the integration of an HIV-1 CA mutant (R264K) that evades the antiviral effects of cytotoxic T lymphocytes (173). Particularly, CypA depletion increased integration of the R264K mutant viral DNA, suggesting a role of CypA in post-nuclear entry steps of infection. Considering the recent reports that intact or almost intact HIV-1 capsid could enter the nucleus (156, 158, 159), it could be predicted that CypA remains engaged with the viral replication complex after nuclear entry and thus could affect nuclear events of infection. However, future studies are needed to clarify these highly speculative models and pinpoint the intra-nuclear role of CA and CA-binding host factors during the early stages of HIV-1 infection. Recently, Xue et al. reported that HIV-1 CA interacts with the nucleoporins NUP35 and POM121 and that the knockdown of these two nucleoporins diminishes infectivity (174). Interestingly, the multi-fold reduction in infectivity imposed by NUP35 or POM121 depletion was rescued by CsA treatment or by the depletion of CypA. The infectivity defect arising from the depletion of these NUPs was suggested to be critically dependent on the interaction between CA and CypA. Collectively, these findings suggested that CypA could block HIV-1 infection when viral access to the nuclear entry pathway regulated by NUP35 or POM121 is impeded, thus underscoring the critical role of CypA in optimal nuclear entry of HIV-1.

Finally, an intra-nuclear role for CA is also supported by infection studies of several HIV-1 CA mutants showing alterations in proviral integration efficiency and integration site selection. For example, the T54A/N57A mutations in CA render HIV-1 infection cell cycle-dependent and map to post-nuclear entry events, including integration targeting (64, 65). Additionally, the infectivity defect of the CsA-dependent CA mutants A92E and G94D has been attributed to impaired chromosomal integration (175, 176). The altered integration efficiency and integration site preferences rendered by the N74D mutation in CA (85, 169, 170) and the retargeted integration by the CypA/RanBP2-independent CA mutant P90A (47, 85) also support an intranuclear role for CA during HIV-1 infection.

#### Shielding HIV-1 DNA from nuclear sensors

The early steps of HIV-1 infection involve a number of viral nucleic acid intermediates. For instance, the reverse transcription of the viral ssRNA genome generates dsRNA, ssDNA, DNA/RNA hybrids, and dsDNA (28). Additionally, the reverse-transcribed viral dsDNA undergoes 3′-processing to produce 5′-overhang and recessed 3′-DNA ends (29). Despite the presence of these viral nucleic acid substrates in an infected cell, chronic HIV-1 infection fails to trigger a strong innate immune response (177). There is evidence that the viral capsid could shield the RNA genome and the RTC/PIC-associated viral DNA from cellular sensors such as cGAS in the cytoplasm (178–186). Interestingly, a recent report suggests that the host factor NONO binds to HIV-2 CA associated with the nuclear viral replication complex and facilitates cGAS-mediated sensing of the viral DNA in the nucleus (182). NONO was identified as a binding partner of both HIV-1 and HIV-2 CA using a yeast-two hybrid system and was shown to form a complex with the nuclear cGAS but not with the cytosolic cGAS. This study reported the presence of HIV-2 CA in the nucleus and suggested that the nuclear CA could mediate HIV-2 innate sensing. However, the mechanism by which NONO regulates the nuclear localization of cGAS and how cGAS senses the nuclear-PIC-associated viral DNA remains unclear. It is also not known whether a similar mechanism exists for HIV-1 CA and cGAS-mediated sensing of HIV-1 DNA in the nucleus.

#### CA-targeting inhibitors decrease HIV-1 integration

HIV-1 CA is a validated anti-viral drug target (187, 188), and several small molecules have been reported to inhibit HIV-1 infection by specifically targeting CA (189, 190). Studies of four inhibitors, PF74, GS-CA1, GS-6207, and coumermycin A1 (C-A1), strongly support the role of CA in the post-nuclear entry steps of HIV-1 infection.

PF74 (PF-3450074) is one of the most-studied and best-characterized CA-targeting antiviral compounds (179, 191, 192). PF74 binds to a pocket at the NTD–CTD inter-subunit interface that is also targeted by CPSF6 and Nup153 (52, 58). PF74 exhibits a concentration-dependent inhibitory effect on HIV-1 replication. For example, at concentrations higher than 2 µM, PF74 inhibits reverse transcription and the nuclear entry step of infection (52, 192, 193). Notably, PF74 triggers premature capsid uncoating at concentrations of 10 µM and above (58, 193, 194). Studies from our laboratory showed that the antiviral activity of PF74 at lower concentrations (<2 µM) (193) maps to the inhibition of HIV-1 integration (123). We observed that at 2 µM, PF74 did not inhibit reverse transcription but reduced nuclear entry up to 50%. Interestingly, the level of reduction in nuclear entry did not account for the >95% inhibition in HIV-1 infection, indicating that PF74 also blocked a post-nuclear entry step or steps. Interestingly, we observed that PF74 significantly reduced proviral integration, and this reduction correlated quantitatively with antiviral activity. To the best of our knowledge, this was the first report of a CA-inhibitor blocking HIV-1 integration step. We also measured the integration activity of cytoplasmic PICs extracted from PF74-treated cells. PF74 markedly inhibited PIC-associated integration activity. This inhibitory effect was specific since PF74 treatment did not reduce the integration activity of PICs of the drug-resistant (5Mut) HIV-1. These data provided further support that inhibition of HIV-1 integration by PF74 is dependent on CA. Finally, there is also evidence that PF74 alters HIV-1 integration site preference (195). Since PF74’s effect is dependent on its binding to CA, collectively, these results strongly support an intra-nuclear role for CA.

GS-CA1 and GS-6207 are highly potent CA inhibitors that bind to the same pocket as PF74 (196–198). GS-CA1 is more potent than PF74 and exhibits three concentration-dependent inhibition mechanisms (196). GS-CA1, at high concentrations (25 nM), interfered with reverse transcription. At intermediate concentrations (5 nM), it did not alter reverse transcription but reduced integration and 2-LTR circles, suggesting that GS-CA1 can prevent nuclear import. Interestingly, at a lower concentration of 0.5 nM, GS-CA1 reduced proviral integration without affecting reverse transcription or nuclear entry, in accordance with our study of PF74 (123). The block to integration in the absence of a concomitant increase in 2-LTR circles suggested that low GS-CA1 concentrations may simultaneously interfere with both nuclear entry and integration. GS-6207 is the second-generation CA inhibitor and the first-in-class long-acting ultra-potent CA inhibitor (197). GS-6207 also showed three concentration-dependent antiviral mechanisms of action (198). At a high concentration of 50 nM, GS-6207 inhibited reverse transcription, whereas the intermediate concentration of 5 nM partly impaired viral DNA synthesis and blocked the formation of 2-LTR circles and integration of HIV-1 DNA. Interestingly, at 0.5 nM, GS-6207 inhibited integration without affecting reverse transcription. These inhibitory effects are consistent with the model that HIV-1 CA is involved in post-nuclear entry steps, including viral DNA integration.

Finally, the antiviral effect of C-A1 has been mapped to HIV-1 integration, and the inhibitory mechanism may depend on the binding of the compound to CA (144, 199). C-A1 has a dual antiviral effect by inhibiting viral DNA integration and viral gene expression. Notably, C-A1’s inhibitory effect on HIV-1 DNA integration is not due to reduced nuclear entry or reverse transcription. Passaging HIV-1 in the presence of C-A1 selected an escape mutant with the A105S mutation in CA. Interestingly, molecular docking analysis and biochemical studies identified a binding pocket of CA-1 in the hexameric HIV-1 capsid. Therefore, the anti-viral effect of this compound has been suggested to be dependent on CA.

Collectively, these studies of CA -inhibitors provide evidence that CA is involved in the post-nuclear entry steps of HIV-1 infection and is functionally linked to the HIV-1 integration step.

### Conclusions and future perspective

The HIV-1 capsid has now emerged as a key player in establishing productive viral infection, not just a container that protects and delivers the viral RNA genome to the target cell. The capsid offers an optimal environment for reverse transcription, shields the viral DNA from cellular sensors, and serves as a functional vehicle for transport toward, into, and within the nucleus. Recent findings by several groups, including ours, have overturned the long-standing view that HIV-1 CA plays a limited role after the nuclear entry step of HIV-1 infection. It is now generally accepted that HIV-1 CA plays an essential role in all the key early steps of infection leading up to viral integration. While the positive roles of CA in reverse transcription, cytoplasmic trafficking, nuclear import, intra-nuclear trafficking, and integration site selection continue to be cemented by new findings, CA’s direct role in viral integration remains an open and contested question. The recent finding that the intact HIV-1 capsid enters the nucleus offers an exciting and important area of research to further define the functional role of CA in the nucleus of an infected cell. We envision that novel and cutting-edge approaches will eventually shed more light on CA’s role in viral integration, thus enabling a consensus view on the topic and, consequently, invigorating interest in pursuing novel CA inhibitors as antiviral drugs.
